# Associated factors of professional burnout among faculty members of graduate *stricto sensu* programs in language teaching and linguistics: a cross-sectional study

**DOI:** 10.1590/1516-3180.2021.1027.R1.21072022

**Published:** 2022-09-12

**Authors:** Maynara Fernanda Carvalho Barreto, Maria José Quina Galdino, Eloiza Rodrigues Vidal de Oliveira, Frederico Garcia Fernandes, Júlia Trevisan Martins, Maria Helena Palucci Marziale, Sonia Silva Marcon, Maria do Carmo Fernandez Lourenço Haddad

**Affiliations:** I PhD. Professor, Department of Nursing, Universidade Estadual do Norte do Paraná (UENP), Bandeirantes (PR), Brazil.; II PhD. Professor, Department of Nursing, Universidade Estadual do Norte do Paraná (UENP), Bandeirantes (PR), Brazil.; III Nursing Student, Universidade Estadual do Norte do Paraná (UENP), Bandeirantes (PR), Brazil.; IV Litt. D. Professor, Department of Vernacular and Classical Letters., Universidade Estadual de Londrina (UEL), Londrina (PR), Brazil.; V PhD. Professor, Department of Nursing, Universidade Estadual de Londrina (UEL), Londrina (PR), Brazil.; VI PhD. Professor, Department of Nursing, School of Nursing, Universidade de São Paulo (USP), Ribeirão Preto (SP), Brazil.; VII PhD. Professor, Department of Nursing, Universidade Estadual de Maringá (UEM), Maringá (PR), Brazil.; VIII PhD. Professor, Department of Nursing, Universidade Estadual de Londrina (UEL), Londrina (PR), Brazil.

**Keywords:** Burnout, psychological, Burnout, professional, Education, graduate, Faculty member, Language teaching, Linguistic teaching, Burnout syndrome

## Abstract

**BACKGROUND:**

The burnout syndrome can be avoided and/or have its signs and symptoms reduced by knowing the five associated factors that help identify the health and working conditions of the professors of graduate programs.

**OBJECTIVE:**

To analyze the factors associated with burnout among faculty members of graduate stricto sensu programs.

**DESIGN AND SETTING:**

A cross-sectional study was conducted among 585 faculty members of Graduate Programs in Language Teaching and Linguistics in Brazil.

**METHODS:**

Data were collected through an online questionnaire. The outcomes were the dimensions of burnout and its related factors identified through multiple templates of logistic regression.

**RESULTS:**

Faculty members with increased chances of emotional exhaustion and depersonalization mentioned the use of medications due to labor activities. The negative influence of pace and intensity of work, thoughts about quitting the program, and having to produce three or more scientific articles were associated with higher chances of emotional exhaustion, while having to achieve nine hours per week in undergraduate programs was related to reduced personal accomplishment. Having a conjugal relationship, satisfaction with health and work, post-doctoral degree, autonomy, and good interpersonal relationships with faculty members of the program reduced the chances of emotional exhaustion. Reduced chances of depersonalization occurred among those who were satisfied with work, had good interpersonal relationships with advisees and faculty members, and received productivity funding.

**CONCLUSION:**

Sociodemographic, health, and occupational factors related to the dimensions of burnout were identified.

## INTRODUCTION

Work, defined as an essential activity and a constituent of human identity, as well as stressful conditions inherent to it, have been associated with imbalances and psychosocial phenomena such as the development of burnout syndrome as an aftermath of transformations, demands, and intensification of the labor process in several professional categories and organizations.^1,2,3^

Burnout syndrome is defined as a phenomenon characterized by a set of signals and symptoms arising from a chronic reaction to interpersonal emotional stressors at work that results in exhaustion or depletion of the worker.^4,5^

The syndrome is a result of the interconnectedness of aspects from the work environment and individual characteristics, in which the phenomenon is pointed out as an issue that affects mainly workers, who have direct contact with other people as part of their job, such as faculty members.^4^

Burnout is a singular occupational phenomenon affecting the worker's health since it refers specifically to the occupational context and is not used to describe experiences in other areas of life, according to the definition in the new International Classification of Diseases (ICD-11) on January 1, 2022.^6^

Burnout affects workers as their emotional resources are reduced, showing signals and symptoms related to emotional exhaustion, depersonalization, and reduced personal accomplishment.^4,5^

Emotional exhaustion is a response characterized by the worker's impoverishment of physical and emotional resources and generally includes the first signals and symptoms related to burnout, such as chronic fatigue sensations and emotional distress. When it appears, workers have minimal physical and emotional resources and m ay feel insufficiently energetic to face new problems or demands related to work.^5^

Depersonalization represents the imbalances related to the interpersonal context of burnout and, when present, workers mention insensibility in their interpersonal relations and excessive disinterest in several aspects related to work. On the other hand, reduced levels of personal accomplishment refer to self-evaluation of burnout once the phenomenon includes several cognitive-attitudinal variables, such as reduced personal accomplishment at work.^5^

Burnout also results in high costs to the organizations and employers. Thus, it is conceivable that there is a need to comprehend the complexity between the ‘self’ of the worker and the context of work, as greater the difference or incompatibility between worker-work contexts, the greater the probability of emotional exhaustion or depletion.^5^

In teaching, factors related to the work environment and conditions, and the organization of work are considered possible agents of health and psychological imbalances of faculty members who work in undergraduate^3,7–8^ and graduate programs.^9^

Postgraduate professors’ work-related processes and conditions can result in their dissatisfaction and health problems,^9^ since the activities performed have their own complexity due to the specificities of the work process and high workload.^10^

However, there are no studies on burnout and its associated factors among faculty members of *stricto sensu* graduate programs in Language Teaching and Linguistics in Brazil, characterizing the uniqueness of this investigation.

Once the phenomenon studied is avoided and/or its signals and symptoms are reduced,^5^ knowing the factors associated with burnout can enable the identification of the work conditions of institutions in which faculty members of graduate programs work, their health conditions, and support can be provided for the development of strategies and assertive interventions within the organizational context.

This study addresses the following research question: “What are the associated factors related to burnout dimensions among faculty members of graduate *stricto sensu* programs in Language Teaching and Linguistics in Brazil?”. This study hypothesized that burnout dimensions were associated with sociodemographic, health, occupational, and lifestyle variables.

## OBJECTIVE

The objective of this research was to analyze the dimensions of burnout and explore the factors associated with it among faculty members of graduate stricto sensu programs in language teaching and linguistics in Brazil.

## METHODS

### Design of the study, description of the sample and places

A cross-sectional study was conducted with 585 faculty members of Graduate Programs in Language Teaching and Linguistics (GPLTL).

The inclusion criteria was permanent faculty members registered on the GPLTL in 2018. Faculty members who were on any sort of leave during 2018 were excluded from this study.

### Measurements

To locate the faculty members, a manual search was conducted between January and April 2019 on *Sucupira Platform*.^11^ To collect the contact information of each faculty member, the information available on each program's website and a list of e-mails received from the program coordinators were used.

A total of 2,270 professors belonging to GPLTL were identified. To define the study sample, the formula n≥50+(8×m) (m: number of independent variables) was used to test multiple associations.^12^ Thus, the minimum number of 370 participants (50+(8×40)) was obtained based on 38 sociodemographic, health, occupational, and lifestyle variables, and the dimensions of burnout.

### Procedures

Data were obtained between February and August 2019 on a virtual platform called Mubble. This platform had a semi-structured questionnaire^13^ that included items of sociodemographic, health, lifestyle, and occupational variables, especially the typical elements of a faculty member's working process. All questions were mandatory to answer to minimize possible losses or failures during data collection.

The information collected was imported into a database enabling statistical analysis of the following variables: marital status (yes/no), health satisfaction (yes/no), usage of medication due to labor activities (yes/no), having a post-doctoral degree (yes/no), time working on undergraduate courses (in years), autonomy at work (yes/no), work satisfaction (yes/no), negative influence of the pace and intensity of work on life (yes/no), thoughts about quitting the GPLTL (no or sometimes/frequently), number of research articles being developed (≤ 2 or ≥ 3), relationship with faculty members from the current GPLTL (bad/regular or excellent/good), having productivity funding (yes/no), working hours in undergraduate teaching (in hours per week), number of books and/or journal chapters published during the last year (≤ 2 or ≥ 3), and the relationship with the advisees (bad/regular or excellent/good).

Burnout syndrome was evaluated using the Maslach Burnout Inventory™ - Human Services Survey (MBI™-HSS), composed of 22 items distributed in three dimensions: emotional exhaustion (nine items), depersonalization (five items), and reduced personal accomplishment (eight items). The responses were measured on a seven-point Likert scale, ranging from 0 to 6.^14^

To analyze the responses, the three dimensions must be analyzed and considered equally as a construct, with the objective of identifying the potential of development of the syndrome and not as a diagnosis of it.^14^ Therefore, when an individual showed higher scores of emotional exhaustion and depersonalization and lower scores on professional accomplishment they might have higher chances of developing the burnout syndrome.^4^

### Statistical analyses

SPSS version 20.0 (IBM, Chicago, Illinois, United States) was used for the data analyses. The outcomes of this study were the dimensions of the MBI™-HSS and the associated factors identified through multiple logistic regression models. In this step, univariate analysis was used to verify the relationship between the dependent and independent variables (sociodemographic, health conditions, and occupational characteristics).

The *stepwise forward* method was used during the multiple model elaboration, in which the independent variables were inserted individually following the order of decreasing values of significance, established by existing research. The final models of regression showed variables with statistical significance of P < 0.05 according to Wald's test and adjusted for gender and age variables, considering that previous works indicated these aspects to be controlled,^15^ as well as years of teaching experience at an undergraduate level and as a senior professor, once they are considered as possible misleading factors in the data analysis.

The variation explained in terms of outcomes was verified using the Nagelkerke R-Squared test. The results were presented as raw odds ratios (OR) and adjusted with 95% confidence intervals (CI).

### Ethical considerations

The study followed the ethical standards, including an approval from the Research Ethics Committee under the legal opinion no. 2,347,839, CAAE:79006017.0.0000.5231. Dated: October 25, 2017.

## RESULTS

This study included teachers from all regions of Brazil. Of the 585 participants, 396 (67.69%) were married. Regarding health satisfaction, 209 (35.73%) were not satisfied, and 278 (47.52%) mentioned the usage of medication due to symptoms they believed was related to their labor activity.

Regarding the educational qualification and teaching experience, 339 (57.95%) faculty members had a post-doctoral degree, 85 (14.58%) received productivity funding, 508 (86.84%) mentioned professional autonomy, 490 (83.76%) were satisfied with their work, and 467 (79.83%) were satisfied with their performance within the GPLTL. However, 345 (58.95%) participants mentioned that they had already thought about quitting activities related to the GPLTL.

Regarding interpersonal relationships, 106 (18.12%) reported not having a good relationship with their colleagues, and 392 (67.01%) believed that the pace and intensity of work negatively influenced their lives.

The participants who informed the usage of medications due to the effect of their labor activity, negative influence of pace and intensity of work in their personal life, thoughts on quitting GPLTL, and having three or more scientific articles being produced, showed increased chances of emotional exhaustion. Reduced chances of emotional exhaustion were shown by faculty members who were in a marital relationship, satisfied with their health, had a post-doctoral degree, autonomy, work satisfaction, and a good interpersonal relationship with their colleagues, as shown in Table 1.

**Table 1.** Multiple model with factors associated with emotional exhaustion among faculty members of graduate stricto sensu programs in Language Teaching and Linguistics. Brazil, 2020

Multiple model	P value	Odds ratio^raw^ (95% CI)	P value	Odds ratio^adjusted*^ (95% CI)
Marital status (yes/no)	**0.011**	1 0.524 (0.319–0.861)	**0.013**	1 0.533 (0.325–0.877)
Health satisfaction (dissatisfied/ satisfied)	**< 0.001**	1 0.411 (0.256–0.660)	**< 0.001**	1 0.419 (0.261–0.675)
Usage of medication due to labor activities (yes/no)	**< 0.001**	1 2.189 (1.403–3.416)	**< 0.001**	1 2.278 (1.448–3.585)
Post-doctoral degree (yes/no)	**0.020**	1 0.584 (0.371–0.919)	**0.030**	1 0.598 (0.376–0.951)
Autonomy at work (yes/no)	**0.014**	1 0.519 (0.308–0.875)	**0.016**	1 0.526 (0.312–0.887)
Work satisfaction (yes/no)	**< 0.001**	1 0.365 (0.223–0.599)	**< 0.001**	1 0.363 (0.221–0.596)
Negative influence of the pace and intensity of work on life (yes/no)	**< 0.001**	1 9.416 (5.432–16.320)	**< 0.001**	1 8,406 (4.737–14.914)
Thoughts on quitting the GPLTL (no or sometimes/frequently)	**0.002**	1 2.054 (1.297–3.252)	**0.003**	1 2.047 (1.284–3.264)
Research articles produced (≤ 2 × ≥ 3)	**< 0.001**	1 2.146 (1.371–3.361)	**< 0.001**	1 2.082 (1.326–3.269)
Relationship with most of the faculty members from the program (bad/regular or excellent/good)	**0.049**	1 0.671 (0.469–0.960)	**0.030**	1 0.672(0.469–0.963)

*Adjusted for gender, age, being a senior professor, and years of teaching experience at an undergraduate level; Nagelkerke R Square:0.553.

CI = confidence interval; GPLTL = Graduate Programs in Language Teaching and Linguistic.

Faculty members who mentioned the use of medications related to their labor activities showed an increased chance of high depersonalization. On the other hand, those who mentioned being satisfied with work, having good interpersonal relationships with advisees and other faculty members, and having productivity funding showed decreased chances of depersonalization (Table 2).

**Table 2.** Multiple model with factors associated with depersonalization among faculty members of graduate stricto sensu programs in Languages and Linguistic. Brazil, 2020

Multiple model	P value	Odds ratio^raw^ (95% CI)	P value	Odds ratio^adjusted*^ (95% CI)
Usage of medication due to labor activities (yes/no)	**0.011**	1 2.033 (1.173–3.525)	**0.011**	1 2.066 (1.179–3.622)
Work satisfaction (yes/no)	**0.004**	1 0.425 (0.239–0.756)	**0.005**	1 0.434 (0.243–0.774)
Relationship with most of the advisees (bad/regular or excellent/good)	**< 0.001**	1 0.312 (0.184–0.530)	**< 0.001**	1 0.321 (0.189–0.547)
Receives productivity funding (yes/no)	**0.022**	1 0.312 (0.115–0.845)	**0.052**	1 0.365 (0.132–1.011)
Relationship with most of the faculty members from the program (bad/regular or excellent/good)	**< 0.001**	1 0.455 (0.304–0.679)	**< 0.001**	1 0.452 (0.302–0.675)

*Adjusted for gender, age, being a senior professor, and years of teaching experience at an undergraduate level; Nagelkerke R Square:0.296.

CI = confidence interval.

With respect to reduced professional accomplishment, Table 3 shows that satisfaction with work and GPLTL, good relationships with advisees and faculty members, and presentation of more than three books and/or publication of journal chapters during the last year decreased the levels of reduced professional accomplishment. Faculty members with a workload of more than nine hours per week in undergraduate teaching demonstrated associations with higher chances of reduced professional accomplishment.

**Table 3.** Multiple model with factors associated with reduced professional accomplishment among faculty members of graduate stricto sensu programs in Languages and Linguistic. Brazil, 2020

Multiple model	P value	Odds ratio^raw^ (95% CI)	P value	Odds ratio^adjusted*^ (95% CI)
Work satisfaction (yes/no)	**< 0.001**	1 0.337 (0.201–0.565)	**< 0.001**	1 0.341 (0.203–0.572)
Workload (hours) of teaching undergraduate courses (≤ 8 × ≥ 9)	**0.006**	1 1.872 (1.196–2.929)	**0.005**	1 1.907 (1.209–3.007)
Relationship with most of the advisees (bad/regular or excellent/good)	**0.007**	1 0.560 (0.366–0.855)	**0.008**	1 0.561 (0.366–0.861)
Satisfaction with the GPLTL (yes/no)	**< 0.001**	1 0.381 (0.222–0.657)	**< 0.001**	1 0.374 (0.215–0.648)
Number of published book and/or journal chapters during the last year (≤ 2 × ≥ 3)	**0.008**	1 0.507 (0.306–0.840)	**0.015**	1 0.533 (0.321–0.885)
Relationship with most of faculty members from the program (bad/regular or excellent/good)	**< 0.001**	1 0.577 (0.411–0.810)	**< 0.001**	1 0.566 (0.402–0.796)

*Adjusted for gender, age, being a senior professor, and years of teaching experience at an undergraduate level; Nagelkerke R Square:0.334. CI = confidence interval; GPLTL = Graduate Program in Language Teaching and Linguistics.

## DISCUSSION

The profiling of the participants showed a predominance of faculty members with marital relationships, which was associated with reduced levels of emotional exhaustion. Although burnout is strongly identified and significantly associated with situational and occupational factors, identifying individual characteristics of the workers is also important and essential to comprehend the development of the syndrome.^4,16,17,18,19^

Regarding the dimensions and associated factors of burnout, it is evident in this study that faculty members who have mentioned the use of medications related to symptoms arising from their professional activities, negative influence from the pace and intensity of work on personal life, thoughts about quitting the program, and publication of three or more scientific articles, showed higher chances (P < 0.05) of emotional exhaustion.

Largely, the emotional exhaustion within faculty members comes from their responsibilities, duties, and tasks in which the workload is increased based on the need to guarantee quality education during the training of new professionals.^20^ It is also associated with the achievement and continuous participation in research, management, and interpersonal relations in the workplace. Emotional exhaustion results in lower capability and/or lower levels of internal resources of emotional intelligence and empathy, attitudes from cynicism, depersonalization, professional inefficiency, sleeplessness, and other health imbalances,^21^ which may explain the factors identified.

Most of the time, faculty members endured unfavorable situations in their workplace due to individual and/or collective protections, avoiding becoming sick to keep working.^22^ In the case of teaching practice in graduate programs, a study reflects upon the hypotheses that should be discussed regarding the work and health conditions of professors.^23^ One of which is that the work of faculty members in graduate programs and the related activities such as tutoring, reporting, managing journals, examining committee, and attending seminars, when intensified, might harm their health.

The other hypotheses referred to the submission of work and productivity ethics as possible causes of becoming sick. Moreover, the social perspective of the incapacity of a faculty member to work while dealing with a personal problem was considered one of the factors that aggravated health,^23^ physical, and mental conditions even more.

It is worth stressing that amongst the faculty members’ activities recommended by Coordination of Improvement of Higher Education Personnel (Coordenação de Aperfeiçoamento de Pessoal de Nível Superior [CAPES]) to a graduate *stricto sensu* professor, there are those related to teaching within the program's courses, development of research and gathering resources from funding agencies, leadership of research groups, tutoring of doctoral and master's students, participation in in undergraduate thesis and scientific initiation of students from other degrees, elaboration of research papers, emission of reports to scientific journals as a reviewer, and working on administrative and undergraduate teaching activities, among other activities.^24^

A study conducted with 550 faculty members from northern Italy found that work overload and conflicts with colleagues and students were positively associated (P < 0.05) with emotional exhaustion. Beyond that, excessive demand from students was considered a stressful factor for faculty members.^25^

Regarding the negative influence of the pace and intensity of work on a person's life, the intense pacing of work was one of the factors that faculty members were exposed to, and when associated with a higher level of stress, showed variations in life quality and health imbalance.^26^

As an example of imbalances related to health, signals and symptoms related to occupational factors such as burnout stand out, and may affect the psycho-affective, musculoskeletal, nervous, digestive, dermatological, cardiovascular, and urogenital systems.^27^ In faculty members, symptoms such as vocal fatigue due to extensive use of voice, hoarseness, dysphonia, and musculoskeletal pain are considered health issues resulting from their working process,^7^ which may justify the use of medications due to professional activities. In this study, faculty members who mentioned the use of medications due to their professional activities also showed 2.066 times higher chances (P = 0.011) of depersonalization due to exhaustion and emotional overload.^5^

Regarding thoughts on quitting the GPLTL, a study^28^ affirms that faculty members show increasing demotivation and emotional distress. It reveals a need for the organizations’ managers to think about actions and strategies to identify the levels of exhaustion of their workers^5^ similar to the ones identified in this study.

Aggravation of the professors’ physical and mental health conditions is among the possible causes for them leaving the profession and/or related activities. Hence, it is essential to concentrate efforts on workers who are generally asymptomatic and healthy,^5^ and improve their capacity of work.^7^ From this aspect, adoption of burnout intervention programs may become strategies for preventing and/or reducing the signals and symptoms of the syndrome and also broadening their effects on GPLTL at the individual, collective, and organizational levels, with the potential to implement changes in the workplace.

Concerning the amount of research in preparation as a risk factor for emotional exhaustion, it stood out that Brazil, the country in which the study was developed, published more than half of all scientific production registered in 2018 in Latin America and showed productivity standards being constantly changed due to academic production goals related mainly to the graduate level.^23^

The criteria to evaluate a faculty member's work and production are mostly associated with a capitalist historical model that permeates college education institutions. Physical and emotional distress related to faculty members’ production occurs from a numerical evaluation of non-measurable aspects, such as the quality of the accomplished activities.^29^ Moreover, it is noted that occupational factors such as quantitative demands, workload, number of working hours, low autonomy during the working process, and absence of social support are strongly related to distress and emotional exhaustion as a response to stress.^30–31^

In contrast, marital status, satisfaction with health and work, autonomy at work, and good relationships with most of the program's faculty members were protective factors (P < 0.05) against emotional exhaustion as found in this study.

It is noteworthy that the level of wellness of a worker influences the quality of work. In this sense, the significance received by workers based on their professional activities, beneficial interpersonal relations in the workplace, and social support are the factors that contribute to the acknowledgment of their identity, motivation, satisfaction, and professional efficiency,^29^ minimizing the emotional exhaustion and risk of burnout, which potentializes engagement at work.^25^

Individual factors such as marital status, motivation, and self-efficacy should also be considered as improvers or protectors to stress and the phenomena that might be triggered by it, such as burnout. Thus, identifying positive occupational aspects, having satisfactory relationships with colleagues, having flexibility in terms of working hours, coexisting and sharing of experiences with students, having autonomy, and the being capable of expansion and construction of knowledge are factors that improve the feeling of pleasure and satisfaction at work^22^ and minimize the signals and symptoms of burnout syndrome.

Being satisfied with the work, having a good relationship with the majority of the program's faculty members and advisees, and receiving productivity funding were associated with lower levels of depersonalization (P < 0.05). However, satisfaction in teaching varies according to the organization of work and its conditions.^7^

Faculty members who mentioned having more than nine hours of undergraduate teaching showed higher chances (P < 0.05) of development of reduced professional accomplishment. From this result, it is defended that high workload is among the main factors of negative influence in the professional life of faculty members, who work on GPLTL, by showing intense working hours within undergraduate courses, validating the note on how performing faculty members’ activities most of the time is challenging. Furthermore, the faculty members’ work does not constitute a workload of more than nine hours for demands and activities, which might result in reduced professional accomplishment.

The number of hours worked, especially the extra hours, is considered a factor that influences fatigue and injury risks, and interferes with motivation and self-evaluation of the professional accomplishment of workers. In cases of a reduced sense of professional accomplishment, and lack of institutional resources, social support and opportunities for professional development, it is possible to intensify perceptions about faculty members’ identity and professional competence, productivity, working hours, and achievement.^5^

A study about work and health conditions among faculty members and high school teachers in Mendoza, Argentina, showed that faculty members with higher professional qualifications demonstrated higher levels of emotional and physical distress arising from demands and conditions they endured at work.^32^ On the other hand, it was highlighted that being satisfied with work at the GPLTL, having good relationship with most of the faculty members, and having at least three books and/or journal chapters published within the last year were protective factors of reduced professional accomplishment.

Hence, it stands out that burnout has, as possible consequences, implications on: personal as well as interpersonal relationships between workers and colleagues, physical health, family issues, increasing usage of substances, and higher chances of depression and suicide ideation.^5^

Once present, besides the development of feelings and negative perceptions as personal anguish,^33^ workers may mention dissatisfaction with their professional achievements,^34^ physical exhaustion, sleeplessness, and adversities with family and interpersonal relationships.^16^ It happens from the imbalance of one or more areas of professional life that are centrally associated with burnout such as, workload, control, rewards, community, justice, and values.^4^

It was observed that due to the uniqueness of the research conducted, studies that did not show differentiation between the levels of teaching in which faculty members worked constituted a limitation that could be the base of the discussion on the findings. In addition, the cross-sectional establishment of this study allowed the identification of associated factors, but not the causal nexus between these factors and the dimensions of higher levels of burnout. Moreover, any type of leave was also considered a limitation because knowing the health and work conditions of faculty members who were off work on medical leave could enhance this investigation. However, the advancement of knowledge through the study of this phenomenon in a group of workers has rarely been explored.

The results support the implementation of strategies at the organizational and individual levels to prevent and/or reduce symptoms related to the three burnout dimensions in different GPLTLs in Brazil.

## CONCLUSION

Sociodemographic, health, and occupational factors related to the dimensions of burnout were identified in this study. The participants who informed the usage of medications due to their labor activity, negative influence of pace and intensity of work on their personal life, thoughts on quitting GPLTL, and having three or more scientific articles being produced, showed increased chances of emotional exhaustion. Faculty members who mentioned the use of medications due to their labor activities showed an increased chance of high depersonalization. Faculty members with a workload of more than nine hours per week in undergraduate teaching demonstrated associations with higher chances of reduced professional accomplishment.
